# Innovative Nursing Care for Patients with Psychological Disorders: A Comprehensive Review on Iranian Versus Global Nursing Interventions

**DOI:** 10.31661/gmj.v13i.3378

**Published:** 2024-08-11

**Authors:** Parvin Malek Mohammadi, Ahmed Hussein Zwamel, Fatemeh Kyani, Maryam Zeydani, Roya Dokoohaki, Soudabeh Behzadi, Rafat Rezapour-Nasrabad, Mohammad Nabizadeh

**Affiliations:** ^1^ Department of Nursing, School of Nursing and Midwifery, Shahid Sadoughi University of Medical Sciences, Yazd, Iran; ^2^ Department of Medical Laboratory Technique, Medical Technique College, The Islamic University, Najaf, Iraq; ^3^ Department of Medical Laboratory Technique, Medical Technique College, the Islamic University of Al Diwaniyah, Al Diwaniyah, Iraq; ^4^ Department of Medical Laboratory Technique, Medical Technique College, The Islamic University of Babylon, Babylon, Iraq; ^5^ Department of Midwifery, Astara Branch, Islamic Azad University, Astara, Iran; ^6^ Department of Nursing, School of Nursing and Midwifery, Ahvaz Jondishapur University of Medical Sciences, Ahvaz, Iran; ^7^ Community Based Psychiatric Care Research Center, Nursing and Midwifery School, Shiraz University of Medical Sciences, Shiraz, Iran; ^8^ Department of Nursing, Arsanjan Branch, Islamic Azad University, Arsanjan, Iran; ^9^ Department of Psychiatric Nursing and Management, School of Nursing and Midwifery, Shahid Beheshti University of Medical Sciences, Tehran, Iran; ^10^ Department of Psychology, Isfahan University, Isfahan, Iran

**Keywords:** Psychological Disorders, Innovative Nursing Care, Mental Health, Evidence-based Practices

## Abstract

Psychological disorders (PDs) are one of the most important global challenges that affect a large number of individuals regardless of geographical location. In Iran, similar to other parts of the world, the prevalence of PDs is increasing and requires innovative nursing care (NC) interventions. The present study aims to comprehensively assess the current NC practices for patients with PDs, as well as compare traditional and innovative approaches between Iran and global healthcare systems. Traditional NC practices in Iran emphasize a holistic and culturally sensitive approach, which integrates traditional NCs with evidence-based therapies; however, global practices often focus on evidence-based and cognitive-behavioral therapies. Innovative NC approaches, e.g., technology-assisted interventions and personal care programs, play a fundamental role in improving treatment outcomes and quality of life for patients with PDs. Also, Iranian nurses are integrating complementary therapies with evidence-based practices to provide comprehensive and personalized care for patients. On a global scale, there is an increasing emphasis on collaborative care models and cutting-edge technologies to enhance the availability and effectiveness of care. Previous evidence indicated the roles of technology-based interventions, concentration techniques, and personal care programs in improving Iranian patient outcomes. While there are challenges such as limited resources, cultural stigma, and lack of specialties both in Iran and around the world, preventive efforts to address these barriers can facilitate the widespread adoption of innovative national NCs. Future research directions include remote psychiatry, integration of complementary therapies with traditional NCs, and providing interdisciplinary collaboration to enhance patient-centered care for individuals with PDs. Hence, by promoting knowledge exchange and collaboration among healthcare professionals, organizations can create a supportive environment for implementing and maintaining innovative NCs that are ultimately able to provide comprehensive mental health care for individuals around the world.

## Introduction

Currently, psychological disorders (PDs) are a significant public health concern that affects individuals, families, and the entire community [1][2]. In Iran, the prevalence of PDs is increasing, caused by factors such as urbanization, industrialization, and the impact of modern lifestyles on mental health [3]. According to previous studies in Iran, it is estimated that nearly 23% of the general population may experience a type of PDs in their lifetime, the most common of which includes depression, anxiety, and substance abuse [4][5]. Regarding the World Health Organization, it is estimated that regardless of geographical location and/or cultural context, about 450 million people worldwide suffer from mental or behavioral disorders [6][7]. In addition, the global burden of PDs is significant and has major impacts on quality of life (QoL), productivity, and overall well-being [8]. Common PDs worldwide include depression, anxiety disorders, bipolar disorder, schizophrenia, and substance abuse [9][10].

In Iran, factors such as stigma, limited access to mental health services, and the decline of qualified mental health professionals are among the most important health-related challenges [11]. Therefore, to reduce PDs, efforts to raise consciousness and lessen the related stigma, and also to increase the global networks in mental health must be put in place [12]. The increasing burden of PDs which varies from one country to another but affects individuals and communities at large is a reminder that there is an urgent need for preventative measures, early interventions as well as other approaches of curative nature [13]. For example, countries may share best practices and work together to improve their mental healthcare system and the outcomes of people with PDs [14]. Currently; only a few studies have been conducted on innovative NC in Iran [15][16], necessitating a comprehensive study that not only explores dimensions, benefits, and barriers associated with patient care with PDs but also compares innovative versus traditional NC.

Hence, our study aimed to provide a brief and comprehensive overview of NC, especially innovative NCs for patients with PDs between the Iranian population and around the world.

## Overview of Traditional NCs Practices

The principles of traditional NC are based on empathy and compassion, which are the building blocks of professionalism in health care [17]. Current research shows that traditional NC enhances patient safety, comfort, and well-being through established standards as well as, evidence-based guidelines [18][19][20]. Traditional NC practices cover a lot of responsibilities including physical examination, medication administration, wound dressing, patient teaching, and counseling on medications [19][21].

One of the major features of traditional NC practices is relying on creating meaningful relationships with patients based on trust, respect, and understanding [22]. For instance, Kwame et al. demonstrated that strong nurse-patient relationships were associated with high levels of patient satisfaction, treatment compliance, and health outcomes [23]. In fact, by providing empathic care and attention to the patients; nurses can establish a safe environment where they feel valued [24]. Therefore, this aspect of the interpersonal component in traditional NC plays an important role in recovery and overall well-being among patients.

Traditional NC practices in Iran for patients with PDs often involve a friendly approach that includes cultural beliefs, family participation, and spiritual healing [25]. In effect, some Iranian nurses may use traditional approaches such as narration, music therapy, herbal remedies, and religious ceremonies as part of managing PDs among their patients [25][26][27]. In other words, these acts consider not only the symptoms and signs of the disorder but also emphasize the feeling side of maladies like emotions, socials as well as spirituality [27][28].

Indeed, traditional NC practices in Iran within PDs focus more on a culture-sensitive approach to mental health management [29][30]. Although Western practices often focus on evidence-based treatments, medication management, and cognitive-behavioral therapies [29][30], Iranian traditional practices tend to incorporate a wider range of modalities that consider the interconnectedness of mind, body, and spirit [31]. This model considers cultural context; social support systems and spiritual convictions when dealing with individuals suffering from PDs [32][33]. On the other hand, there are studies that attempted to evaluate the complementary aspects of certain major mental health interventions that revealed that NC’s indices could further efficacies of therapeutic outcomes for Iranian PDs [28][33]. Therefore, drawing on both traditionally established therapies and evidence-based therapies tends to enhance treatment engagement, cultural competence, and patient satisfaction in various international populations [34][35][36][37].

## Innovative NC approaches

**Table:** Table1. Comparison of Most Important Aspects of NCs Between Iran and Worldwide

Aspect of NCs	Iran	Worldwide
Cultural sensitivity	Emphasis on incorporating cultural norms, religious beliefs, and customs into NC interventions to enhance patient engagement and treatment outcomes tailored to Iranian culture	Recognizes the importance of cultural competence in delivering mental healthcare, with a focus on understanding and respecting the cultural backgrounds of patients to provide individualized and effective care across diverse populations.
Technology integration	Incorporating telepsychiatry, digital mental health platforms, and mobile health applications to expand access to mental health services, especially in underserved areas and rural communities.	Utilizing technology to deliver innovative mental health interventions globally, with a focus on leveraging telehealth, virtual reality, smartphone apps, and online cognitive-behavioral therapy to enhance access, convenience, and effectiveness of mental healthcare delivery.
Research and collaboration	Encouraging collaborative research efforts between academic institutions, healthcare organizations, and government agencies to promote innovation, knowledge exchange, and capacity building in mental health nursing.	Fostering research partnerships and networks on a global scale to support interdisciplinary research initiatives, evidence-based practice, and continuous learning in mental health NC worldwide, with a focus on translating research findings into clinical practice and policy development.
Training and education	Enhancing nursing education programs with a focus on mental health nursing competencies, training, and workshops to equip nurses with the knowledge and skills necessary to provide high-quality care for patients with PDs.	Promoting ongoing professional development, training, and education for nurses globally to ensure competency in evidence-based practices, innovative interventions, and the latest advancements in mental healthcare delivery to meet the evolving needs of patients with PDs.
Patient-centered care	Promoting a patient-centered approach in NC delivery for individuals with PDs, focusing on empathy, active listening, and empowering patients to participate in their treatment decisions.	Advocating for patient-centered care models globally that emphasize holistic, personalized care, shared decision-making, and collaboration between patients, families, and healthcare providers to improve treatment outcomes, enhance patient satisfaction, and promote recovery in individuals with mental health conditions.

The different innovative NCs contribute to the treatment and management of patients with PD, and nurses carry the massive responsibility of supporting and caring for people with mental health issues. Therefore, it can be concluded that there is substantial potential for enhancing patient’s outcomes and QoL through the application of innovative solutions [38]. Another thing that creatively enhances the application of innovative NCs for patients with PD is Technology-Assisted Interventions [39].

Indeed, telehealth services, mobile apps, and online platforms help nurses offer patients remote monitoring, counseling, and education resulting in increased patients’ access to care and self-management promotion [40].

Currently, innovative NCs involve the use of evidence-based practices including cognitive behavioral therapy, mindful practices, and dialectical behavioral analysis the patient care programs [41]. These therapies are useful in treating different types of PDs, helping the patients acquire adaptive strategies to manage their symptoms and enhance their QoL [42][43]. Thus, the nurses who are trained in these approaches draw individualized interventions for every patient and, consequently, enhance the level of empowerment and functionality in the process of healing [44].

Besides, the innovative NCs involve having a more encompassing standpoint on mental health, its socio-factors, culture, and patients’ tendencies [45]. Also, an individual-centered care approach is practiced by nurses to allow patients to participate in decision-making, goal setting, and planning of the treatment plan [46]. In other words, the aspect of health can be discussed physically, emotionally, socially, and even spiritually.

As stated, the diverse health-related issues enable the nurses to assist patients with PD in dealing with the challenges fully and optimize their QoL [47].

In this regard, Iranian nurses apply complementary therapies (music therapy, art therapy, and mindfulness techniques) with scientific evidence in developing creative NCs [48]. Consequently, they are capable of offering holistic and individualized treatment that encompasses the psychological, emotional, and spiritual realms of well-being [48].

In this context, NCs programs for patients with PD are also experiencing significant developments on an international level to optimize the therapeutic approach’s efficacy and benefits. There has been a shift in the focus on team practice and nurses are expected to work together with psychiatrists, psychologists, social workers, and many other members of the healthcare for the development of care plans more efficiently [49]. Furthermore, nurses employ recent technologies like virtual reality (VR), cognitive behavioral therapy via digital platforms, and home monitoring systems to enhance both the arability and quality of care for patients with PD [50]. Table-1 shows the main differences in innovative NC strategies between nurses in Iran and around the world. Although there may be different ways of implementing these strategies in Iran compared to other countries, the fundamental principles of patient-centered care (PCC), evidence-based practice, and collaboration remain the same [51].

Specific innovative approaches

For patients with PDs, different methods in NCs aim at improving outcomes and patients’ QoL [52]. For instance, technology-based interventions have greatly advanced the arena of mental health by providing new ways through which individuals with PDs may access therapy and support [53]. Also, VR therapy enables artificial exposure of the patient to certain scenarios as a way of treating certain phobias or traumatic experiences [54]. Telepsychiatry services avail mental healthcare through remote evaluation and treatment thus being useful in areas where patients can easily access mental health professionals [55].

Another relatively new method that is widely incorporated in NCs plans for treating PDs is mindfulness techniques [56]. These practices focus on cultivating the awareness of the current moment, the attitude of acceptance, and non-judgment and are effective in decreasing such states as stress, anxiety, and depression symptoms [56][57]. Nurses are prescribing mind-ﬁnite approaches (including guided relaxation, breathing techniques, and scanning) to enhance patients’ self-awareness, and their emotional management, and coping skills in response to their mental disorders [58].

It is crucial for patients with PDs to have treatment that is tailored to their specific preferences and needs to promote active involvement in their care and their recovery [59]. Tailoring may involve collaborating with patients to create customized therapy sessions, adapting communication styles according to patient needs, and working with patients to form attainable and measurable objectives for their care [60][61].

## Evidence-Base and Effectiveness of Innovative NCs

Empirical evidence supporting innovative NCs

Regarding Jamali et al. [62] through evidence-based research showed how some approaches to innovative NCs have the potential to improve the quality of mental health care and patient outcomes [62]. There are also some studies [63][64] from Iran that indicated technology-based interventions (e.g., smartphone applications that track mood, or online psychotherapy platforms) have been used and appreciated by patients with PDs and have improved outcomes through the reduction of symptom severity and adherence to treatment [63][64]. For example, previous studies in Iran have shown mindfulness-based interventions, like mindfulness-based stress reduction (MBSR) programs or mindfulness meditation, have been incorporated into NCs plans and are beneficial for patients with PDs [65][66]. Also, using mindfulness-based interventions during and after the NC has been successful, and there is the potential for these interventions to facilitate emotional well-being and resilience [67].

The available evidence from Iran suggests that personalized mental health care plans in NCs can be more effective and lead to better outcomes and increased patient satisfaction [68]. By considering the individual patient’s needs, preferences, and cultural factors, it is believed that the care plan nurses implement are promoting the feelings of empowerment and involvement in their treatment [69]. This conversation is consistent with international best practice, which states that PCC is a key part of providing quality mental health services [70]. The international trends in the available evidence from Iran provide important insights into this innovative approach [71]. Despite the cultural and contextual characteristics that may change the way that these are applied, technology, mindfulness and care plans tailored toward the individual appear to be concepts that promote and benefit the delivery of mental health care [71][72]. To further investigate and apply these innovative practices in nursing practice in Iran and worldwide is extremely important, and the result could be the continued evolution of the mental healthcare field and better outcomes for patients with PDs [73].

Evaluation of the effectiveness and outcomes of innovative NCs approaches

Limited evidence has shown the potential of innovative NCs in improving PDs treatment outcomes among Iranian patients [74]. For example, Farsi et al. [75] indicated that new applications of technology (e.g., smartphone apps and online psychotherapy platforms) could be effective in symptom reduction, adherence to the treatment process, and patient engagement [75]. Also, other studies revealed that mindfulness interventions could significantly affect anxiety, depression, and stress for PDs patients [76][77]. In fact, the integration of mindfulness techniques in NCs plans has led to an improved emotional regulation capacity, better coping skills, and good QoL [78]. Results are supported by international works [79] that demonstrated the effectiveness of mindfulness practices as a scientific consensus across populations worldwide resource to support mental well-being as well as resilience against various emotional disturbances. Furthermore, the evaluation of personalized care plans in Iran has shown favorable outcomes in enhancing patient satisfaction, treatment adherence, and holistic recovery [80].

A comparison of these findings with those from global trends shows that evaluation of innovative NCs interventions in patients with PDs has had equal positive results and benefits [81][82]. In other words, evidence is accumulating in support of the effectiveness of technology-based interventions, mindfulness interventions, and personalized care plans as good practice examples. We can use these new approaches based on our cultural situation and healthcare setting [83]. So, by sharing best practices and evaluation results in Iran or a few other countries related to this issue, we can contribute to evidence-based advancement care practices for people with different PDs worldwide [84].

## Challenges and Barriers

Identification of challenges in implementing innovative NCs

In Iran, the major issue the health system faces are the poor infrastructure and resource challenges in relation to the implementation of technology-based interventions in the treatment settings for mental disorders [85]. The establishment of works on novel NC models that rely on digital tools to drive improved patient outcomes would not be successful with no predetermined access to secure internet services available, training healthcare practitioners on the use of the digital platform, and reliable funding for the technological investments [86][87].

Cultural beliefs and stigma on mental health may lead to increasing implementation challenges with innovative NC for patients with PD [87]. In fact, further states that misconceptions about mental illness, traditional attitudes towards the help-seeking process, and social taboos all could make barriers to the acceptance of modern care approaches within the health system [88]. Hence, reducing these cultural barriers is possible through concentrated education, awareness, and advocacy initiatives to reduce stigma and provide a more supportive environment for individuals with PDs [89]. Similarly, the challenges of implementing new NCs for PD patients worldwide reflect some of the issues faced in Iran. Limited funding and resources, gaps in access to mental health services, regulatory barriers, and lack of trained staff shortages may further impede the adoption and sustainability of new models of care globally [90]. Also, differences in health care, reimbursement policies, and regulatory frameworks in different countries make it difficult to provide greater standardization of alternative NC strategies [91].

Moreover, such a lack of collaboration and communication within the healthcare team raises barriers to organized holistic management of care for patients with PDs [91]. As such, the need to break barriers between health providers becomes real in Iran and globally to enhance interdisciplinary teamwork and collaborative practice when caring for patients to ensure innovative NCs deal with increasing demands that are complex among the population with various mental health problems [92]. By recognizing and addressing these challenges, nurses can work on overcoming the present barriers brought forward by the implementation of innovative NC approaches and, hence, improve outcomes among patients with PDs [92]. Moreover, such a lack of collaboration and communication within the healthcare team raises barriers to organized holistic management of care for patients with PDs [91]. As such, the need to break barriers between health providers becomes real in Iran and globally to enhance interdisciplinary teamwork and collaborative practice when caring for patients to ensure innovative NCs deal with increasing demands that are complex among the population with various mental health problems [92]. By recognizing and addressing these challenges, nurses can work on overcoming the present barriers brought forward by the implementation of innovative NC approaches and, hence, improve outcomes among patients with PDs [92].

Barriers to the widespread adoption of NCs

The barriers to transform the mental health care system into an innovative NCs approach for patients with PDs are multilayered and cause resistance to change [93]. For example, in Iran, a main limiting factor is the lack of trained mental health professionals working with this population and special training programs in PD management [94]. An inherent limitation of evidence-based practice across mental health conditions is the availability of a workforce with the competence in delivering innovative NCs designed around the unique needs of individuals with these conditions, which tends to decline the scale-up and sustainability of such approaches within the health system [95]. Financial barriers and inadequate funding for mental health services in Iran also prevent the widespread adoption of new NCs for PD patients [96]. Limited budget allocation, competing health care priorities, factors of ineffective allocation in the implementation of new care models, access to necessary equipment and technology, and inadequate mental health program financial support and long-term commitment from policymakers may inhibit the investment path to effectively reach the recipient population [97]. Hence, in the absence of specialized mental health services, the integration of alternative NC strategies remains a challenge in Iran [97].

The obstacles to the widespread introduction of alternative NC strategies to PD patients worldwide include similar challenges faced in Iran, such as differences in access to mental health services, legal barriers, and inadequate resources to support technological advances in care delivery [98]. Furthermore, cultural norms, language barriers, and social stigmas related to mental health issues may prevent further implementation of care used in different parts of the world [98]. So, identifying and addressing these common barriers may serve to remove barriers to the dissemination of alternative NC strategies for PD patients through healthcare systems, in Iran and more responsive and patient-centered global healthcare can provide perspective [99].

## Future Directions and Recommendations

A key research direction is to examine the effectiveness and feasibility of telepsychiatry and telehealth services in providing additional NC to PD patients. Examining the impact of remote care, virtual counseling, and digital mental health tools to provide independent patient care better penetration, treatment adherence, and clinical outcomes may provide insights.

There are also alternatives to provide complementary and alternative holistic care, such as mindfulness-based interventions, art therapy, and animal-assisted therapy, combined with traditional NC techniques in patients with PD can be provided. Indeed, the combined effects of innovative NCs and complementary strategies to reduce symptom severity, enhance QoL, and improve individuals with mental illness study of falls can inform evidence-based guidelines and clinical practice recommendations for healthcare professionals [100].

In addition, multidisciplinary collaboration involving nurses, psychiatrists, psychologists, social workers, and community health workers is essential to promote a coordinated and integrated approach to care for patients with PD [101]. A model of team-based care to promote communication, continuity of care, and treatment effects.

Studies examining the impact of team-based care models, care coordination strategies, and interprofessional education programs to improve communication, continuity of care, and clinical outcomes could consider as best practices for integrating new NC into routine practices in a mental health setting.

Empowering the health professional through knowledge and skills development in collaboration and sharing creates an enabling environment for innovative approaches in NCs for widespread to patients with PDs to accomplish improved patient outcomes and holistic mental health care delivery. Further research and recommendations to include additional NC in routine practice for patients with PD, especially in Iran, may enhance the delivery of mental health services, and improve outcomes for individuals with mental health conditions. Also, an important research direction is to identify cultural interventions and evidence-based practices that are consistent with Iranian socio-cultural norms.

Investigation on the effectiveness of culturally sensitive NCs—like the incorporation of religious beliefs, values, and customs in psychotherapeutic interventions— may improve engagement and adherence to mental health services for Iranian patients with PDs.

## Conclusion

The comparison of alternative NC approaches for PD patients between Iran and the rest of the world highlights the importance of understanding and respecting cultural differences. Integrating cultural sensitivity into mental health in Iran, effectively using technology, has promoted collaborative multicultural research. A key focus is on cultural competence around the world, technology integration, research programs, education and training programs, and patient care to improve the delayed mental health outcomes of individuals with PD.

Indeed, recognition of uniqueness, strengths, and differences in NC approaches between Iran and the global community allows mutual learning, best practices, and continuous improvement to be exchanged in mental health nursing. These innovations, improvements, and PCC in the scope of nursing practice are to be taken with priority, this might hold golden opportunities throughout the world for increased quality care delivery, better accessibility to services, and better health outcomes for people coping with psychological challenges. Areas, including diversity, technology, research, and education are some of the most potent for promoting innovations and sustainable improvement in mental health NC across an international scale.

## Conflict of Interest

All the authors declare there are no any conflicts of interest.
